# Unmet Healthcare Needs and Healthcare Access Gaps Among Uninsured U.S. Adults Aged 50–64

**DOI:** 10.3390/ijerph17082711

**Published:** 2020-04-15

**Authors:** Namkee G. Choi, Diana M. DiNitto, Bryan Y. Choi

**Affiliations:** 1 The University of Texas at Austin Steve Hicks School of Social Work, Austin, TX 78712, USA; ddinitto@mail.utexas.edu; 2 Brown University Warren Alpert Medical School, Department of Emergency Medicine, Providence, RI 02903, USA; bchoiemdoc@gmail.com

**Keywords:** near-older adults, uninsured, private and public health insurance, healthcare access

## Abstract

Lack of health insurance (HI) is a particular problem for near-older Americans aged 50–64 because they tend to have more chronic health conditions than younger age groups and are at increased risk of disability; however, little recent research has focused on HI coverage and healthcare access among this age group. Using the U.S. National Health Interview Survey data for the years 2013 to 2018, we compared HI coverage and healthcare access between the 50–64 and 65+ age groups. Using logistic regression analysis, we then examined the sociodemographic and health characteristics of past-year healthcare access of near-older Americans without HI to those with private HI or public HI (Medicare without Medicaid, Medicaid without Medicare, Medicare and Medicaid, and VA/military HI). We estimated the odds of healthcare access among those without HI compared to those with private or public HI. Near-older Americans without HI were at least seven times more likely to have postponed or foregone needed healthcare due to costs, and only 15% to 23% as likely to have had contact with any healthcare professional in the preceding 12 months. Expanding HI to near-older adults would increase healthcare access and likely result in reduced morbidity and mortality and higher quality of life for them.

## 1. Introduction

Americans aged 50–64 constitute almost 20% of the U.S. population [1]. This age group, often referred to as “near-older adults,” is at substantial risk of multiple chronic health conditions and disability. For example, the 2014 National Health Interview Survey data showed that 32.1% of the 45–64 age group, compared to 7.3% of the 18–44 age group, had two or more chronic health conditions (including arthritis, asthma, cancer, chronic obstructive pulmonary disease, coronary heart disease, diabetes, hepatitis, hypertension, stroke, or weak/failing kidneys) [2]. The working-age population (18–64 years) with chronic conditions has significantly higher odds of experiencing an uninsured period and reporting healthcare access barriers than those without chronic conditions [3,4]. Obtaining health insurance (HI) and healthcare is difficult for many Americans, because the United States is the only developed country that lacks universal health coverage. According to the Organisation for Economic Co-operation and Development (OECD), universal health coverage improves health outcomes, including life expectancy, and provides financial protection against impoverishing health care costs [5].

Americans aged 65+ do have near-universal public HI through Medicare, a federally administered social insurance program. Medicare receipt is usually based on an individual’s (or spouse’s) prior payroll tax contributions. Younger Americans generally do not have access to Medicare, even if they have made payroll tax contributions for many years, unless they have received Social Security Disability Insurance (SSDI) for at least 24 months, have been diagnosed with End-Stage Renal Disease, or began receiving SSDI for amyotrophic lateral sclerosis [6]. 

In addition to Medicare, the other major U.S. public HI program is the means-tested, federal- and state-funded Medicaid program, which serves many low-income Americans regardless of age and work history. All U.S. states have a Medicaid program, and under the 2010 Patient Protection and Affordable Care Act (also known as the ACA or “Obamacare”), 36 states and the District of Columbia expanded Medicaid coverage to more low-income adults; however, 14 states did not, leaving many non-disabled adults aged 18–64 without Medicaid access [7]. The U.S. Veterans Health Administration (VA) also provides healthcare coverage for veterans (and their dependents or survivors in some cases). The proportion in the 65+ age group who have VA healthcare coverage is higher than that in the 50–64 age group. There is other healthcare coverage (e.g., Tricare) for active duty and retired members of the uniformed services, their families, and survivors.

More than two-thirds of the working-age population in the United States has private HI, mostly through their employer [8]. However, most people who are not working or whose employer does not sponsor HI must purchase their own private health coverage or go without HI. Prior to the ACA, many more Americans had difficulty obtaining HI in the individual voluntary HI market, because they could not afford the premiums and/or because a preexisting health condition made obtaining coverage more difficult [9]. These barriers were greater for near-older adults, given their higher likelihood of having preexisting conditions compared to younger adults [9]. 

The ACA was the largest HI expansion in the United States, since Medicare and Medicaid were established more than fifty years ago in 1965. Under the ACA, more Americans gained private HI coverage due to (1) a mandate that all employers with 50+ employees offer insurance to their employees or pay a penalty; (2) a federally-operated Health Insurance Marketplace or state-based exchanges where adults can compare private HI plans and purchase a plan; (3) provision of a premium tax credit (for those with household income between 100% and 400% of the federal poverty level) and cost-sharing subsidy (to reduce out-of-pocket spending for deductibles and co-payments among those who are eligible to receive a premium tax credit, and have a household income between 100% and 250% of poverty); and (4) an individual HI coverage mandate (although the penalty for not having coverage was reduced to zero as of 2019) [9,10]. 

The National Health Interview Survey data showed that the proportion of uninsured adults aged 18–64 decreased between 2013 and 2015 [11]. The decrease was especially notable among the 45–64 age group, and largely attributable to the ACA [9,12]. However, the proportion of the uninsured began increasing again in 2017, due to several changes to the ACA under the Trump Administration [13,14]. Unsubsidized premiums increased substantially between 2017 and 2018, in part due to the Trump Administration’s decision to cease payments to insurers for cost-sharing reductions [15,16]. In 2018, 13.3% of the 18–64 age group did not have any HI; the rates were higher among those who are poor or near-poor and among non-Hispanic Blacks and Hispanics, compared to non-Hispanic Whites [17,18]. The uninsured rate is likely to continue to trend upward, now that the ACA’s individual mandate penalty has effectively been rescinded [19]. 

Even with HI, out-of-pocket healthcare expenses can be substantial. Though the out-of-pocket burden for those with chronic conditions (e.g., diabetes) has decreased over time, those who are poor and near-poor still bear a significantly higher burden than middle- and high-income individuals [20]. Given that the poor or near-poor are more likely to lack HI, they may also be more likely to delay or forego healthcare or experience financial hardship, due to high out-of-pocket expenses when they do get care [21]. A study of uninsured adults aged 18–64 based on the 2013 Medical Expenditure Panel Survey found that the uninsured visited emergency departments (EDs) more often than those with private insurance, but, contrary to public perception, they used it substantially less than those insured by Medicaid, and had a significantly lower rate of hospital admissions than those with any HI [22]. 

Although research has identified the negative health and financial effects of a lack of HI among the 18–64 age group, we found little recent research on healthcare access gaps among uninsured near-older adults. Given the large number of near-older U.S. adults, including many baby boomers, it is important to examine their health coverage status and how the lack of HI may affect their healthcare access. In this study, based on multi-year National Health Interview Survey data, we first examined differences in HI coverage and healthcare access between the 50–64 and 65+ age groups. Then, focusing on the 50–64 age group, we examined: (1) HI coverage and healthcare access, or lack thereof, in years 2013 through 2018, and (2) sociodemographic and health characteristics and past-year healthcare access of those without any HI compared to those with any private HI and four types of public HI (i.e., VA/military HI, Medicare without Medicaid, dual Medicare and Medicaid, and Medicaid without Medicare). We also examined sociodemographic and health characteristics associated with no HI versus any HI. Finally, we fit multivariable models to examine the odds of eight different types of healthcare use/nonuse among those without HI compared to those with different types of HI. Based on previous studies of uninsured adults, study hypotheses were that compared to those in the five HI groups, those without HI would have: (Hypothesis 1) significantly greater odds of delaying healthcare or not receiving healthcare due to cost and significantly lower odds of any contact with healthcare professionals, and (Hypothesis 2) significantly lower odds of any ED visit and overnight hospital stay. The study may provide insight into the needs of near-older adults with limited healthcare access as they age without HI.

## 2. Materials and Methods

### 2.1. Data and Sample

Data came from the 2013–2018 National Health Interview Survey (NHIS) public use data files downloaded from the CDC’s National Center for Health Statistics web site. The NHIS is an annual, cross-sectional household survey, which is the principal source of information on the health, health coverage, and healthcare access of the civilian, noninstitutionalized U.S. population [23]. For each sampled household, interviews were conducted (mostly face-to-face) with an adult family member who answered questions about each family member’s demographic and health status characteristics, health coverage, and healthcare access and use. The NHIS also collects more detailed health and other data from one Sample Adult from each household, which is the primary data source for the present study. Combining six consecutive years of annual NHIS data resulted in 49,293 Sample Adult respondents aged 50 to 64 years and 48,287 aged 65+ (NHIS public use data sets do not provide the chronological age of those aged ≥85 years). In this study, we included 49,025 individuals aged 50–64 and 48,284 aged 65+ after excluding those with missing data on HI coverage. 

### 2.2. Measures

#### 2.2.1. Health Insurance (HI) Coverage

We focused on the following six HI categories (1 = yes; 0 = no): (1) no HI (i.e., uninsured); (2) private HI; (3) VA/military health coverage (referred to as VA/military HI hereafter); (4) Medicare without Medicaid; (5) dual Medicare and Medicaid; and (6) Medicaid without Medicare. In NHIS, the uninsured are those who were not covered by either private or public HI throughout the entire year or survey eligibility period, although any healthcare they did obtain may have been paid for or covered by sources that were not defined as HI coverage, such as the VA, community and neighborhood clinics, the Indian Health Service, state and local health departments, state programs other than Medicaid, workers’ compensation, and other unclassified sources (e.g., automobile, home, or liability insurance) [24]. Individuals with Indian Health Service coverage only are considered uninsured [24]. Those with any private HI are individuals with private insurance that provided coverage for hospital and physician care at any point during the year, including plans available through the Federal Health Insurance Marketplace and state-based exchanges [24]. VA/military HI, Medicare, and Medicaid coverage refers to having any of these types of public HI coverage at any time during the past year. The six HI groups were not mutually exclusive. For example, a small proportion of near-older adults with private HI also had VA/military HI (0.9%) and Medicare (2.4%) with or without Medicaid; and a small proportion (4.6%) of Medicare, with or without Medicaid, beneficiaries also had VA/military HI.

#### 2.2.2. Healthcare Access and Use in the Past 12 months

We used the following eight measures (1 = yes; 0 = no): (1) delay or nonreceipt of needed healthcare due to cost; (2) saw/talked to any healthcare professional; (3) saw/talked to a general doctor/practitioner; (4) saw/talked to a medical specialist; (5) saw a dentist; (6) saw/talked to an eye doctor; (7) any ED visit; and (8) any overnight hospital stay. We also examined the following for descriptive purposes only: whether or not the respondent (1) had a usual place for sickness care; (2) used any medication cost-saving strategy (skipped medication doses, took less medicine, delayed filling a prescription, asked doctor for lower-cost medicine, bought prescription drugs from another country, or used alternative therapies); and (3) worried about paying medical bills for sickness and/or injury.

#### 2.2.3. Sociodemographic Factors

These were: (1) age; (2) gender; (3) race/ethnicity (non-Hispanic White, non-Hispanic Black, Hispanic, Asian, and Other); (4) marital status (not married vs. married); (5) education (no bachelor’s degree vs. bachelor’s degree); (6) ratio of family income to official U.S. poverty threshold (<200%, 200–399%, ࣙ400%, and missing); and (7) work status (did not work at all vs. worked full- or part-time in the past year). 

#### 2.2.4. Health Status

These were: (1) number (0–10) of diagnosed chronic health conditions (arthritis, asthma, diabetes, hypertension, heart disease, stroke, chronic kidney disease, liver disease, lung disease, and cancer) with which the respondent had ever been diagnosed; and (2) any functional limitation (1 = yes; 0 = no), defined as the reported level of difficulty with function and activities due to physical and mental/cognitive health problems, injury, and vision and hearing problems.

### 2.3. Analysis

All analyses were conducted with Stata 15/MP’s svy function to account for NHIS’s stratified, multi-stage probability sampling design, and to ensure that variance estimates incorporate the full sampling design. First, we used *χ^2^* tests to describe and compared the 50–64 age group to the 65+ age group on health coverage and delay or nonreceipt of healthcare, contact with any healthcare professional, having or not having a usual place for sickness care, and worry about paying for sickness/injury care. Second, we used *χ^2^* tests to examine any changes from 2013 through 2018 in the proportions of the 50–64 age group by the six HI coverage statuses. Third, we also used *χ^2^* tests and *t* tests for pairwise comparisons between the no HI group and each of the five HI groups on sociodemographic and health statuses and healthcare access and use. Fourth, we used a logistic regression model to examine sociodemographic and health status correlates of no HI versus any HI. Fifth, and finally, to test study hypotheses on healthcare access, we used 40 logistic regression models to compare odds of each of the eight healthcare access variables between the no HI group and each of the five HI groups. Sociodemographic and health characteristics were controlled in each logistic regression model. Variance inflation factor diagnostics, using a cut-off of 2.50 [25], showed that multicollinearity among covariates was not a concern for any model. Logistic regression results are presented as adjusted odds ratios (AOR) with 95% confidence intervals (CI). Statistical significance was set at *p* < 0.05.

## 3. Results

### 3.1. Health Coverage among the 65+ Age Group and the 50–64 Age Group

Table 1 shows that 9.9% of the 50–64 age group, compared to 0.7% of the 65+ age group, had no HI. Of the 50–64 age group, 69.2% had private HI, 4.2% had VA/military insurance, 6.6% had Medicare, 6.2% had Medicaid, and 2.2% were dual Medicare-Medicaid beneficiaries. As expected, for the 65+ age group, Medicare is the most common HI. In terms of healthcare access, 8.1% of the 50–64 age group, compared to 3.3% of the 65+ old group, reported not having a usual place of care; 12.4% of the 50–64 age group, compared to 4.5% of the 65+ old group, reported delaying or not receiving needed healthcare due to cost; 86.4% of the 50–64 age group, compared to 93.6% of the 65+ age group, reported contact with a healthcare professional; and 47.2% of the 50–64 age group, compared to 29.1% of the 65+ age group, reported worrying about paying medical bills. 

### 3.2. Health Coverage among the 50–64 Age Group from 2013 to 2018

Table 2 shows that the proportions of those who reported delay or nonreceipt of healthcare and no health coverage decreased significantly between 2013 and 2016, but increased in 2017 and 2018. The proportion that reported no health coverage in 2018 (9.4%) is still significantly lower than in 2013 (14.5%). The proportions with private HI increased between 2013 and 2015 and was stable in subsequent years. The proportion of those with Medicaid (without Medicare) increased between 2013 and 2016, but decreased in 2017 and 2018. The proportions of those having VA/military HI and Medicare with/without Medicaid did not change during the study period.

### 3.3. Sociodemographic and Health Characteristics of those without Health Coverage

Table 3 shows that those without HI were younger than those with any type of HI, and included lower proportions of women and non-Hispanic Whites. Almost a quarter of those without HI were Hispanic. More than two-thirds reported working in the past year, but 43.3% had an income <200% of poverty, which was a higher percentage than among those with private HI or VA/military HI. Dual Medicare and Medicaid beneficiaries had the lowest income of all. The uninsured also had the lowest number of chronic health conditions of all and the second lowest proportion (45.6%), after the private HI group (37.7%), of those with any functional limitation. Additional analysis showed that compared to 7.7% of non-Hispanic Whites, 11.8% of Blacks, 20.9% of Hispanics, 8.6% of Asians, and 17.9% of all other races/ethnicities lacked HI. Compared to the 5.1% of those with an income >400% of poverty, 16.1% of those with an income <200% of poverty and 10.9% with an income 200–399% of poverty lacked HI.

With respect to healthcare access, 37.5% of the uninsured reported that they did not have a usual place to seek care if they became sick. The rates were 6.4% and 5.1% for those with Medicaid and private HI, respectively, and <4% for those with VA/military HI and Medicare. More than three-quarters of the uninsured reported worrying about paying medical bills, more than a third used medication cost-saving strategies, and about 60% had any contact with a healthcare professional, which was well below the rates among those with any HI. Contacts with a medical specialist, dentist, and eye doctor were especially low. The proportion of the uninsured who had any ED visit was slightly lower than that of those with private HI but substantially lower than those with public HI. The rate of overnight hospital stays among the uninsured did not differ from the rate among those with private HI, but was substantially lower than rates among those with public HI.

### 3.4. Sociodemographic and Health Correlates of Lack of HI: Multivariable Analysis

Table 4 shows that the odds of not having any HI were lower among older than younger individuals and women than men. The odds were greater among non-Hispanic Blacks (AOR = 1.18, 95% CI = 1.05–1.33), Hispanics (AOR = 2.34, 95% CI = 2.09–2.93), and others (AOR = 2.18, 95% CI = 1.75–2.71) compared to non-Hispanic Whites. The odds were also greater among those who were not married, did not have a college degree, and were living under 200% of poverty (AOR=2.67, 95% CI = 95% CI = 2.38–2.99) compared to those withan income 400+% of poverty, and among those with any functional limitation. The odds of not having any HI were lower among those with higher numbers of chronic medical conditions.

### 3.5. Odds of Past-year Healthcare Access among those without HI Compared to those with HI

Table 5 shows the binary logistic regression results controlling for sociodemographic and health characteristics. Not having any HI was associated with 15 times, more than seven times, and almost five times greater odds of delaying or not receiving healthcare compared to having private HI or Medicaid without Medicare, VA/military HI or dual Medicare and Medicaid, and Medicare without Medicaid, respectively. 

Not having any HI was associated with significantly lower odds of any healthcare professional contact including a general practitioner, medical specialist, dentist, and eye doctor (these odds were as low as 0.15 [95% CI = 0.12–0.18] and 0.16 [95% CI = 0.11–0.22], compared to those with Medicare and dual Medicare and Medicaid, respectively, and 0.23 [95% CI = 0.20–0.23] and 0.23 [95% CI = 0.19–0.28], compared to private HI and Medicaid without Medicare, respectively). Among those without any HI, the odds of any ED visit were lower compared to those with any public HI but did not differ from those with private HI, and the odds of any overnight hospital stay were significantly lower compared to those with private or public HI. Findings support both hypotheses.

## 4. Discussion

This study found that almost one in ten (or about six million) Americans aged 50–64 had no HI in 2018, with rates significantly higher than this among those with low-income and racial/ethnic minorities. Of the uninsured aged 50–64, more than 40% who reported needing health care in the past year reported that they delayed or did not receive care due to costs, and only 60% had any contact with a healthcare professional. Although the 2018 rate of uninsured near-older adults is lower than the nearly one in seven without HI in 2013, the lack of HI among a significant portion of near-older adults can have significant health and financial consequences for them and for the nation as a whole.

The uninsured were younger and had fewer chronic health conditions than those with private or public HI, but a larger proportion had functional limitations compared to those with private HI. Given their functional limitations, chronic health conditions among some uninsured individuals may have been under-detected since they were substantially less likely to see a healthcare provider. Since more than two-thirds of those without HI worked but more than 43% had an income <200% of poverty, many likely had low-paying jobs that did not offer HI. For these near-older adults and the one third who did not work, paying for HI on their own may have been difficult, even with ACA’s premium subsidy. 

Our study also showed that Medicare and/or Medicaid beneficiaries were the sickest and poorest of the 50–64 year olds. This is not surprising, since Americans under age 65 must be disabled to participate in Medicare, and Medicaid serves only those with low income. The study also shows that Medicare and Medicaid are lifelines for near-older adults, as those with Medicare and/or Medicaid had much lower rates of delaying or not receiving healthcare, and higher rates of contact with healthcare professionals than their age peers without any HI. In fact, a study of mortality among near-older adults in states with and without ACA Medicaid expansion found that, from 2014 to 2017, Medicaid expansion saved the lives of at least 19,200 adults aged 55 to 64, whereas 15,600 people in the same age group died prematurely due to state decisions not to expand Medicaid [26]. Another study found substantial increases in prescription drug utilization under ACA Medicaid expansion, including medications for diabetes, HIV and Hepatitis C, and cardiovascular disease [27]. Medicaid (with or without Medicare) clearly helps significant numbers of African American and Hispanic near-older adults access needed healthcare, underscoring Medicaid’s important role in expanding health care access and reducing disparities in care for those who are more likely to be poor and have more chronic health problems. 

The key study findings are the large gaps in care between those without any HI and those with private or public HI. Controlling for sociodemographic and health statuses, those who lacked HI were seven times more likely to have postponed or foregone needed healthcare due to cost, and only 15% to 23% as likely to have had contact with any healthcare professional in the preceding 12 months. We also found that the uninsured aged 50–64 were less likely to have visited an ED than those with public HI; however, in contrast to an aforementioned earlier study finding [22], we found no difference in ED visits between the privately insured and those without any HI. We also found that near-older adults without any HI were significantly less likely to have had an overnight hospital stay than those with any type of HI. Lower utilization rates may be due to the high cost of healthcare services, as 40% reported delay or nonreceipt of care due to cost. For near-older adults who have difficulty paying for the basic necessities, healthcare costs must be weighed against housing, utilities, and food costs [13]. 

The 60% of uninsured near-older adults who reported any contact with healthcare professionals are likely to have utilized safety-net health systems such as public hospitals, federally qualified health centers and other community clinics, or other local providers. However, these safety-net systems do not close the access gap, and the financial burden associated with illness often falls on the sickest and uninsured/underinsured. Moreover, in areas without safety-net health systems, the uninsured and other vulnerable people may have to utilize non-safety-net providers, which has serious financial implications for healthcare systems, communities, and states [28]. A study of hospital discharges in West Virginia found that uninsured near-older adults were more likely to be admitted for emergency conditions, to have comorbidities, complications, and longer hospital stays, and to incur higher charges (constituting 30% of all uninsured charges) [29]. Their self-pay and charity care charges were also higher than for any other adult age group [29]. 

For one in ten near-older adults, affordable and reliable health coverage remains elusive. They may lack access to affordable, employer-sponsored HI, be ineligible for HI subsidies through the ACA due to their income, or not qualify due to their immigration status. Some may be eligible for premium tax credits but still have difficulty paying for premiums, and others may also be unaware of their healthcare options [13]. With the continuing erosion of the ACA, the rate of uninsured Americans is rising again. For many vulnerable near-older adults, the consequence of not having HI is compromised health from lack of preventive care and timely attention to acute and chronic health conditions. When they are finally able to enroll in Medicare at age 65, many are likely to need expensive care, further straining the resources of the major public HI system for older adults. Their own financial resources may be depleted from personal healthcare spending, causing them to need public assistance programs like Medicaid in old age.

There are some study limitations due to data constraints. First, the NHIS uninsured rate is conservative, since HI coverage was defined as having HI “at any time during the past year.” Some may have had HI for only part of the year. Second, as some categorized as having HI may have had it for only part of the year, associations between HI and healthcare access should be interpreted cautiously. For example, the high rates of ED visits among Medicare and Medicaid beneficiaries may have been due to visits during the time when they did not yet have Medicare or Medicaid. Third, as both HI and healthcare access were self-reported, the reliability and validity of data may have been affected by recall bias. Fourth, since NHIS data are cross-sectional, we can report associations (correlations), but not causal or longitudinal relationships. Fifth, despite the multi-year data, sample sizes for some subgroups are small, and the findings need cautious interpretation. 

Notwithstanding these limitations, the study findings show healthcare access gaps among near-older adults without HI. The OECD believes that, with rapid population aging worldwide, efforts to achieve universal health coverage throughout the world should not be delayed [5]. Despite calls for “Medicare (or Medicaid) for all,” U.S. political, economic, and cultural environments have worked against adopting government-sponsored universal health coverage. Pluralistic approaches, including expanding eligibility for Medicaid and restoring and expanding other ACA provisions (including premium and cost-sharing subsidies and individual mandate penalties) are considered more realistic pathways to increase HI accessibility and affordability for those currently uninsured [12,30]. As discussed, Medicaid expansion for low-income adults has helped to reduce excess morbidity and mortality. Coverage extensions under the ACA have lowered out-of-pocket spending and provided financial risk protections [31]. These provisions are especially important for near-older adults who are less likely to be employed than younger adults. Medicare eligibility for near-older adults needs expanding. Medicare for those receiving SSDI and those with amyotrophic lateral sclerosis and end-stage renal disease has provided healthcare access for millions of people under age 65 [32]. Expanding Medicare eligibility for all near-older adults without other HI would significantly increase healthcare access among this segment of the U.S. population.

## 5. Conclusions

One in ten near-older adults in the U.S. lacks HI. The rate of uninsured Americans decreased in 2014 and 2015, following ACA implementation, but it increased again in 2017 and 2018, as some ACA provisions were retracted. Compared to those with private or public HI, those without any HI were seven times more likely to have postponed or foregone needed healthcare due to cost, and only 15–23% as likely to have had any healthcare professional contact in the past year. To mitigate the negative health and financial impacts for near-older adults who lack HI, further expanding Medicaid, reinstituting ACA provisions, making Medicare available at an earlier age, and/or other approaches for removing financial barriers to obtaining health coverage are needed. 

## Figures and Tables

**Table 1:** Health insurance (HI) coverage, healthcare access, and healthcare cost worry among the 50–64 age group versus the 65+ age group, % (95% confidence interval).

	50–64	65+
N	49,025	48,284
No HI	9.93 (9.53–10.34)	0.65 (0.56–0.75)
Private HI	69.19 (68.51–69.86)	45.77 (44.84–46.70)
VA/military HI	4.17 (3.90–4.45)	8.05 (7.67–8.45)
Medicare without Medicaid	6.61 (6.31–6.93)	81.94 (81.37–82.50)
Medicare and Medicaid	2.18 (2.03–2.35)	6.06 (5.69–6.45)
Medicaid without Medicare	6.21 (5.88–6.57)	1.18 (1.05–1.34)
Had no usual place for sickness/injury care in past 12 months	8.14 (8.07–8.77)	3.28 (3.08–3.49)
Worried about paying for healthcare	47.23 (46.64–48.01)	29.13 (28.52–29.74)
Healthcare delayed or not received due to cost in past 12 months	12.35 (11.97–12.73)	4.52 (4.29–4.76)
Saw any healthcare professional in past 12 months	86.37 (85.93–86.79)	93.60 (93.32–93.87)

All probability values from Pearson χ^2^ tests for age group comparisons were significant at *p* < 0.001.

**Table 2:** The 50-64 age group: Changes in healthcare access and health insurance (HI) coverage status, 2013–2018 (%).

	Healthcare Delay or Nonuse due to Cost in Past 12 Months	No HI	Private HI	VA/Military HI	Medicare	Medicare + Medicaid	Medicaid
Combined N (% of all in 50–64 age group), 2013–2018	7408 (12.35)	5373 (9.93)	31,392 (69.19)	2209 (4.17)	3567 (6.61)	1506 (2.18)	3563 (6.21)
2013	14.09	14.48	67.62	4.19	6.67	2.03	4.45
2014	12.32	11.18	68.68	4.35	6.27	2.19	5.56
2015	11.09	8.13	70.65	4.47	6.82	2.26	6.12
2016	11.54	7.67	70.33	4.16	6.40	2.30	7.21
2017	11.78	8.83	69.96	3.55	6.90	2.34	7.15
2018	13.30	9.41	70.49	4.28	6.63	1.97	6.74
P value, changes, 2013–2018	<0.001	<0.001	0.013	0.271	0.778	0.710	<0.001

Probability values were calculated from Pearson χ^2^ tests.

**Table 3:** Comparison of those without any health insurance (HI) to those with different types of HI among the 50–64 age group.

	No HI	Private HI	VA/Military HI	Medicare	Medicare + Medicaid	Medicaid
N (% of all 50–64 years old)	5373 (9.93)	31,392 (69.19)	2209 (4.17)	3567 (6.61)	1506 (2.18)	3563 (6.21)
Age, M (SE)	56.22 (0.08)	56.75 (0.03)	57.62 (0.12)	58.59 (0.10)	57.19 (0.14)	56.61 (0.10)
% Female	48.56	52.32	36.31	48.61ǂ	56.45	57.35
Race (%)						
Non-Hispanic White	53.87	75.74	68.60	68.85	54.47	46.44
Non-Hispanic Black	13.85	9.39	16.72	15.67	23.15	21.78
Hispanic	24.61	8.66	7.54	10.16	14.44	20.60
Asian	4.27	4.92	3.98	2.69	2.04	7.5
All other	3.41	1.28	3.17	2.63	5.91	3.69
% Not married	45.65	23.94	30.39	45.94	72.55	56.55
% Without college degree	84.78	60.08	33.37	86.19ǂ	94.35	90.40
Income (%)						
<200% of poverty	43.29	17.11	25.11	47.19	66.42	63.16
200–399% of poverty	25.62	23.28	26.31	25.44	15.83	18.13
400+% of poverty	21.21	51.04	40.23	19.85	9.84	11.06
Missing	9.88	8.57	8.36	7.52	7.91	7.65
% Worked in the past year	68.28	82.91	56.03	10.27	7.83	35.50
No. of chronic medical conditions, M (SE)	1.12 (0.02)	1.22 (0.01)	1.85 (0.04)	2.63 (0.04)	3.07 (0.06)	2.16 (0.04)
% With any functional limitation	45.55	37.71	56.32	88.32	92.26	69.74
Healthcare access in past 12 months (%)						
Had no usual place for sickness/injury care	37.47	5.11	3.75	3.84	2.92	6.43
Worried about paying for healthcare	78.96	42.50	27.06	57.98	40.19	48.29
Healthcare delayed or not received due to cost in past 12 months	40.18	7.47	5.56	22.08	11.17	14.0
Saw/talked with						
Any healthcare professional	59.67	88.84	91.92	94.89	95.22	89.80
General practitioner	45.54	77.54	83.22	86.20	85.94	80.02
Medical specialist	12.70	33.19	40.73	54.54	53.80	37.15
Dentist	33.15	75.18	65.17	46.98	39.19	42.96
Eye doctor	20.44	49.42	50.69	44.41	46.56	37.89
Used cost-saving strategy for prescription drugs	34.07	18.92	11.61	39.09	25.87	22.92
No. of emergency department visits						
0	82.0	84.96	73.31	61.84	50.66	62.89
1	10.62	9.97	14.60	18.35	19.23	16.95
2–3	4.33	2.89	8.01	12.34	17.15	11.98
4+	1.64	0.92	3.01	6.37	11.69	6.20
Missing	1.41	1.26	1.06	1.10	1.27	1.98
Overnight hospital stay	6.05	7.03ǂ	11.05	21.78	28.34	17.97

Note: All probability values from Pearson χ^2^ tests for paired comparisons (i.e., no HI vs. private HI; no HI vs. VA/military HI; no HI. vs. Medicare; no HI vs. Medicare+Medicaid; no HI vs. Medicaid) were significant at *p* < 0.05 except those marked with ǂ.

**Table 4:** Sociodemographic and health correlates of no health insurance (HI) among the 50–64 age group.

	No HI vs. Any HI AOR (95% CI)
Age	0.98 (0.97–0.99) ***
Female vs. male	0.82 (0.76–0.88) ***
Race/ethnicity: Vs. Non-Hispanic White	
Non-Hispanic Black	1.18 (1.05–1.33) ***
Hispanic	2.34 (2.09–2.93) ***
Asian	1.15 (0.94–1.41)
Other	2.18 (1.75–2.71) ***
Not married vs. married	1.74 (1.60–1.90) ***
No college degree vs. college degree	2.29 (2.06–2.55) ***
Income: Vs. 400+% of poverty	
Up to 200% of poverty	2.67 (2.38–2.99) ***
200–399% of poverty	1.82 (1.63–2.04) ***
Missing	2.05 (1.77–2.37) ***
Did not work vs. worked full- or part-time	1.05 (0.95–1.16)
No. of chronic medical conditions	0.75 (0.73–0.78) **
Any functional limitation vs. none	1.11 (1.02–1.22) *
N = 49,025; Model statistics: Design df = 907; F (14, 894) = 96.39, *p* < 0.001	

* *p* < 0.05; ** *p* < 0.01; *** *p* < 0.001.

**Table 5:** Past year healthcare access among those without any health insurance (HI) compared to those with different types of HI among the 50–64 age group: Results from binary logistic regression models.

	No HI vs.				
	Private HI	VA/Military HI	Medicare w/o Medicaid	Medicare + Medicaid	Medicaid w/o Medicare
	AOR (95% CI)	AOR (95% CI)	AOR (95% CI)	AOR (95% CI)	AOR (95% CI)
Medical care delay/nonuse due to cost	7.27 (6.52–8.10)	15.48 (12.20–19.65)	4.65 (3.88–5.58)	14.87 (11.14–19.85)	7.20 (6.05–8.57)
Saw any healthcare professional	0.23 (0.20–0.25)	0.18 (0.14–0.22)	0.15 (0.12–0.18)	0.16 (0.11–0.22)	0.23 (0.19–0.28)
Saw general practitioner	0.28 (0.25–0.30)	0.23 (0.19–0.27)	0.23 (0.20–0.28)	0.26 (0.20–0.34)	0.27 (0.23–0.32)
Saw medical specialist	0.32 (0.28–0.36)	0.29 (0.24–0.35)	0.24 (0.20–0.29)	0.27 (0.21–0.35)	0.34 (0.29–0.41)
Saw dentist	0.25 (0.23–0.27)	0.28 (0.24–0.33)	0.41 (0.35–0.49)	0.44 (0.35-0.55)	0.52 (0.45–0.60)
Saw eye doctor	0.34 (0.31–0.37)	0.30 (0.26–0.35)	0.38 (0.32–0.44)	0.30 (0.24–0.38)	0.47 (0.40–0.54)
Visited Emergency Department	1.02(0.92–1.15) ǂ	0.74 (0.62–0.89)	0.68 (0.57–0.81)	0.63 (0.50–0.80)	0.62 (0.54–0.72)
Hospitalized	0.69 (0.57–0.82)	0.78 (0.60–1.01) ǂ	0.51 (0.39–0.65)	0.57 (0.43–0.76)	0.54 (0.43–0.69)

Note: Each cell represents the odds of healthcare delay/nonaccess or the specific type of healthcare access (shown in the first column) for those without any HI compared to those with the specific type of HI (shown in the second row), controlling for age, gender, race/ethnicity, marital status, education, income, work status, number of chronic medical conditions, and any functional limitation (vs. none). All odds are significant at *p* < 0.001 except those marked with ǂ (*p* > 0.050).
